# Nef Neutralizes the Ability of Exosomes from CD4^+^ T Cells to Act as Decoys during HIV-1 Infection

**DOI:** 10.1371/journal.pone.0113691

**Published:** 2014-11-25

**Authors:** Julianne V. de Carvalho, Rodrigo O. de Castro, Elaine Z. M. da Silva, Paola P. Silveira, Mara E. da Silva-Januário, Eurico Arruda, Maria C. Jamur, Constance Oliver, Renato S. Aguiar, Luis L. P. daSilva

**Affiliations:** 1 Department of Cell and Molecular Biology, Ribeirão Preto Medical School, University of São Paulo, Ribeirão Preto, Brazil; 2 Molecular Virology Laboratory, Department of Genetics, Federal University of Rio de Janeiro, Rio de Janeiro, Brazil; German Primate Center, Germany

## Abstract

Nef is an HIV-1 accessory protein that promotes viral replication and pathogenesis. A key function of Nef is to ensure sustained depletion of CD4 and MHC-I molecules in infected cells by inducing targeting of these proteins to multivesicular bodies (MVBs), and ultimately to lysosomes for degradation. Nef also affects cellular secretory routes promoting its own secretion via exosomes. To better understand the effects of Nef on the exocytic pathway, we investigated whether this viral factor modifies the composition of exosomes released by T lymphocytes. We showed that both CD4 and MHC-I molecules are secreted in exosomes from T cells and that the expression of Nef reduces the amount of these proteins in exosomes. To investigate the functional role for this novel activity of Nef, we performed *in vitro* HIV-1 infection assays in the presence of distinct populations of exosomes. We demonstrated that exosomes released by CD4^+^ T cells, but not CD4^−^ T cells, efficiently inhibit HIV-1 infection *in vitro*. Because CD4 is the main receptor for HIV-1 infection, these results suggest that CD4 molecules displayed on the surface of exosomes can bind to envelope proteins of HIV-1 hindering virus interaction with target cells and infection. Importantly, CD4-depleted exosomes released by CD4^+^ T cells expressing Nef have a reduced capacity to inhibit HIV-1 infection *in vitro*. These results provide evidence that Nef promotes HIV-1 infection by reducing the expression of CD4 in exosomes from infected cells, besides the original role of Nef in reducing the CD4 levels at the cell surface.

## Introduction

The Human Immunodeficiency Virus types 1 and 2 (HIV-1 and HIV-2) mainly infect T lymphocytes and macrophages/monocytes that express CD4, a cell surface type I transmembrane glycoprotein that is the primary receptor for HIV. Host cell infection begins with the interaction of the gp120 subunit of the viral envelope glycoprotein (Env) complex with CD4. This interaction leads to conformational changes in gp120 that increase its affinity for a co-receptor, usually CCR5 or CXCR4 chemokine receptors. Co-receptor binding to Env triggers additional conformational changes in the gp41 subunit that promote the tethering of the viral envelope to the cell plasma membrane and elicit fusion [1], [2]. The CD4-Env interaction is crucial for viral entry, thus downregulation of CD4 surface expression [3] or the use of competitor molecules that target this interaction [4]–[6] can inhibit HIV-1 infection.

Following host cell infection by HIV, one of the most highly transcribed viral genes encodes the accessory protein Nef [7], a critical determinant for high viral loads and progression from infection to full-blown AIDS [8]–[12]. Nef is a myristoylated, 25- to 34-kDa protein encoded by the HIV-1, HIV-2 and Simian Immunodeficiency Virus (SIV) genomes and is found both free in the cytoplasm and associated with the cytosolic face of cellular membranes [13]. Nef is able to modulate signal transduction and protein trafficking machineries in infected cells [14], [15]. The most prominent functions of Nef are the downregulation of cell surface of CD4 and MHC-I molecules. Nef binds to the cytosolic tail (CT) of CD4 and MHC-I and disrupts the intracellular trafficking of these proteins through different mechanisms [14]. Nef promotes endocytosis of CD4 through clathrin-coated vesicles by forming a tripartite complex with the CT of CD4 and the adaptor protein (AP) complex 2 [16], [17] in clathrin pits at the plasma membrane [18], [19]. In contrast, Nef prevents MHC-I from reaching the plasma membrane by promoting retention of these molecules in the Golgi apparatus via interaction with AP-1 [20]–[25]. Nef can also induce MHC-I internalization indirectly through activation of a signal transduction pathway involving assembly of a kinase cascade [26]–[28]. Importantly, both models support the idea that Nef redirects MHC-I molecules toward late endosomes/multivesicular bodies (MVBs), in the endolysosomal pathway. In fact, upon Nef expression both CD4 and MHC-I are targeted to MVBs [21], [29], [30] and ultimately to lysosomes for degradation [20], [31]–[34].

Nef also promotes changes in endosomal morphology [35] and induces accumulation of MVBs [36], [37] and lysosomes [38]. Alternatively to fusing with lysosomes, MVBs can fuse with the plasma membrane and release their intraluminal vesicles (ILVs) as exosomes. Interestingly, it has been recently shown that expression of Nef induces the release of Nef-containing exosomes [39], [40]. Exosomes released by MVBs are approximately 40–100 nm in diameter and contain typical marker proteins, such as Endosomal Sorting Complex Required for Transport (ESCRT) proteins (e.g., TSG101), ESCRT-associated proteins (e.g., Alix), and tetraspanin proteins (e.g., CD63 and CD81) [41]. Although the composition and function of exosomes have been studied predominantly in dendritic cells, macrophages, T cells, B cells, mast cells and several types of tumor cells, these nanovesicles are thought to be released by most cell types [42]. As a consequence, exosomes are ubiquitously present in bodily fluids and play important roles in intercellular communications in the organism.

Since it has been shown that Nef modulates expression of specific surface proteins and promotes release of exosomes, we examined whether Nef could modify the content of exosomes released from T cells. We show that Nef has the ability to reduce the amounts of both CD4 and MHC-I molecules secreted in exosomes. To investigate a possible physiological role for this novel activity of Nef, we performed *in vitro* HIV-1 infection assays in the presence of exosomes from CD4^−^ and CD4^+^ T cells, and also Nef expressing CD4^+^ T cells. Strikingly, exosomes released by CD4^+^ T cells strongly inhibit HIV-1 infection *in vitro* in a concentration-dependent manner. In contrast, exosomes released by CD4^−^ T cells or CD4^+^ T cells expressing Nef are inefficient in preventing HIV-1 infection. We suggest that Nef may contribute to HIV-1 infectivity by reducing the levels of CD4 receptor in exosomes, thereby neutralizing the inhibitory effect of these extracellular vesicles.

## Materials and Methods

### Cell culture

PEAK cells, which are HEK-293 cells transfected with the large T antigen of SV-40 [43] were kindly provided by Dr. Reuben Siraganian (National Institutes of Health, Bethesda, EUA). The following cell lines were obtained from the NIH AIDS Research and Reference Reagent Program (Germantown, MD): the human A3.01 CD4^+^ T cell line and the A2.01 CD4^−^ T cell line, a clonally selected CD4^−^ mutant of A3.01; both originally deposited by Dr. Thomas Folks [44], [45], and the MT-4 T cell line originally deposited by Dr. Douglas Richman [46], [47]. These cells were cultivated in RPMI 1640 medium (Life Technologies, Carlsbad, CA) supplemented with 100 U/mL penicillin, 0.1 µg/mL streptomycin, 2 mM L-glutamine, and 10% fetal bovine serum (Life technologies) at 37°C with 5% CO_2_. HEK 293-T cells from American Type Culture Collection (Manassas, VA) were maintained in DMEM (Life Technologies) supplemented as described above.

### Plasmids, retroviruses, HIV-1 Luc^+^ reporter production and titration

Retroviral system plasmids pVSV-G, pCL-Eco and pMSCV-IRES-GFP were provided by Dr. Dario S. Zamboni (University of São Paulo, Brazil). To produce the pMSCV-Nef-IRES-GFP vector, the DNA fragment encoding NL4 Nef was transferred from pCIneo-Nef [30] to pMSCV-IRES-GFP plasmid using the *BglII* and *SalI* restriction sites. Retroviruses expressing Nef and GFP or GFP alone were generated by cotransfection of PEAK cells (3×10^6^) in a 100 mm tissue culture dish with: 3 µg pVSV-G, 6 µg pCL-Eco and 9 µg of pMSCV-IRES-GFP or pMSCV-Nef-IRES-GFP, using 55 µL of 25 kDa linear polyethylenimine (PEI) (1 µg/µl stock solution) transfection reagent (Polysciences Inc, Warrington, PA). Cell supernatants containing retroviruses were collected 36 h after transfection and stored at −80°C. HIV-1 luciferase reporter viruses were produced in HEK-293T cells. Briefly, 3×10^5^ cells were transfected in 6 well/plate using pNL4.3 ΔNef Luc^+^ Env^+^ plasmid previously described [48], using Lipofectamine 2000 (Life Technologies) according to the manufacturer's protocol. Supernatants containing viruses were collected 48 hours after transfection and stored at −80°C. HIV-1 titer was determined by p24 levels in supernatants using the RETROtek HIV-1 p24 antigen ELISA kit (ZeptoMetrix Corporation, Buffalo, NY).

### Transduction of T cells for expression of GFP or Nef/GFP

A3.01 cells (3×10^4^) were incubated with supernatant from PEAK cells containing Nef/GFP or GFP retrovirus for 24 hours. Transduced cells were washed with PBS and cultivated in complete medium for 72 hours. GFP-positive cells were sorted using a JSAN Cell Sorter (BAY Biosciences, Kobe, Japan). After sorting, A3.01 cells expressing Nef and GFP (Nef/GFP) or GFP alone (GFP), were expanded in culture and homogeneous GFP expression was confirmed by FACS prior to use in experiments.

### Antibodies

Unconjugated or allophycocyanin (APC)-conjugated monoclonal antibodies to human CD4 (S3.5), used for immunofluorescence and fluorescence-activated cell sorting (FACS) analysis, were from Life Technologies. APC-conjugated monoclonal antibody to human HLA-A2 (BB7.2), used for FACS was from BD Biosciences (San Jose, CA). Rabbit polyclonal antibody to human HLA-A2, used for Western blots was purchased from ProteinTech (Chicago, IL). Rabbit polyclonal antibodies to human CD4 (H-370), CD63 (H-193) and goat polyclonal antibody to human Alix (N-20), used for Western blots were purchased from Santa Cruz Biotechnology (Santa Cruz, CA). Rabbit polyclonal antiserum to HIV-1 Nef was obtained from the NIH AIDS Research and Reference Reagent Program (originally deposited by Ronald Swanstrom) [49]. Rabbit antiserum to HRS and GFP were generous gifts from S. Urbé (University of Liverpool, United Kingdom) and R. Hegde (University of Cambridge UK), respectively. Mouse monoclonal antibodies to EEA1 (clone 14/EEA1) and cytochrome c were from BD Biosciences. Mouse monoclonal antibody to HRS (clone A-5) was from Enzo Life Sciences (Farmingdale, NY). Mouse monoclonal antibody to LBPA (clone 6C4) was from Echelon Biosciences (Salt Lake City, UT). Mouse monoclonal antibodies to human transferrin receptor (clone H68.4) and secondary antibodies conjugated to fluorophores were purchased from Life Technologies. Horseradish peroxidase (HRP)-conjugated donkey anti-mouse IgG and donkey anti-rabbit IgG were from GE Healthcare (Piscataway, NJ). HRP-conjugated rabbit anti-goat IgG was from Jackson ImmunoResearch (West Grove, PA).

### FACS analysis

To determine cell surface antibody binding, A3.01 cells grown in suspension were washed twice with ice-cold PBS and incubated with blocking buffer (PBS, 2% BSA). After that, cells were incubated on ice for 45 min with primary antibodies anti-CD4 or anti-HLA-A2 conjugated to APC. After three washes with ice cold PBS the cells were fixed in PBS containing 4% paraformaldehyde (PFA). GFP fluorescence was used to identify and select transduced cells. Corresponding isotype control IgGs conjugated to APC were used as negative controls. The levels of APC fluorescence in cells expressing GFP were measured with a BD FACSCanto II flow cytometer (BD Biosciences) and analyzed using the FlowJo software v7.6.5 (Treestar, Ashland, OR).

### Immunofluorescence microscopy

A3.01 T cells expressing GFP or Nef/GFP were adhered to glass slides coated with Biobond – Tissue Adhesive (Electron Microscopy Sciences, Hatfield, PA). The cells were fixed with 4% PFA in PBS for 15 min at room temperature and then permeabilized with 0.2% Triton X-100 in PBS for 10 min. Cells were then incubated with primary antibody diluted in blocking solution (0.2% pork skin gelatin in PBS), washed in PBS and incubated with secondary antibodies diluted in blocking solution. Coverslips were mounted using Fluoromount G (EM Sciences). Images were acquired using a Leica TCS SP5 (Leica Microsystems, Wetzlar, Germany) confocal microscope.

### Purification of exosomes and preparation of total cell lysates

To allow for comparative analysis, in each experiment, exosomes were collected from equal amounts of culture media conditioned by an equivalent number of cells. Exosomes were purified as described previously [50]. Briefly, cell culture media was prepared using fetal bovine serum (FBS) depleted of exosomes by ultracentrifugation at 100,000×*g* at 4°C for 1 hour, followed by filtration through a 0.22 µm filter. Cells (2×10^5^ cells/mL) were cultured in complete medium free of contaminating vesicles for 72 h and after this period the cells were pelleted by centrifugation at 2,000×*g* for 10 min. The supernatant was passed through a 0.22 µm filter to remove cell debris and the filtrate was ultracentrifuged at 100,000×*g* for 1 h at 4°C. The pellet containing exosomes was collected and washed twice by ultracentrifugation with PBS. The resulting pellet was used directly for electron microscopy or infection assays. For Western blots, exosomal pellets were solubilized with 50 µl of SDS-PAGE sample buffer (4% SDS, 160 mM Tris-HCl [pH 6.8], 20% [vol/vol] glycerol, 100 mM DTT and 0.1% bromophenol blue) and boiled. The cell pellets were washed with ice cold PBS and resuspended in lysis buffer (50 mM Tris-HCl [pH 7.5], 150 mM NaCl, 10% [vol/vol] glycerol, 5 mM EDTA, 1% [vol/vol] Triton X-100) supplemented with protease inhibitor cocktail (Sigma Aldrich, St. Louis, MO) at 4°C, mixed with sample buffer and boiled.

### SDS-PAGE and Western blot analysis

Total cell and exosome lysates were mixed with SDS-PAGE sample buffer, proteins were separated by SDS-PAGE under reducing conditions and electrotransferred to nitrocellulose membranes (Millipore, Bedford, MA). The blots were probed with individual primary antibodies, and then incubated with donkey anti-mouse, anti-rabbit or anti-goat IgG conjugated to HRP. In all blots, proteins were visualized by enhanced chemiluminescence using ECL Plus (GE Healthcare, Piscataway, NJ).

### Transmission electron microscopy

Exosomes were pelleted from 100 mL of cell culture supernatants as described above and the pellet was suspended in 150 µL of PBS. A volume of 30 µL was placed on 15×5 mm pieces of Biobond coated Thermanox coverslips (EM Sciences), incubated for 40 minutes and then washed with 0.1 M cacodylate buffer (pH 7.4). The exosomes were fixed in 2% glutaraldehyde (EM Sciences) and 2% paraformaldehyde (EM Sciences) in 0.1 M cacodylate buffer (pH 7.4) containing 0.025% de CaCl_2_ for 1 hour at room temperature. The exosomes were subsequently washed twice with cacodylate buffer and postfixed in 1% osmium tetroxide (EM Sciences) for 2 hours, rinsed in Milli-Q water, and dehydrated in a graded ethanol series. Alternatively, the pellet from ultracentrifugation containing exosomes was resuspended in 100 µL of binding buffer (PBS with 0.1% BSA) and incubated with 50 µL of magnetic beads coated with anti-CD63 antibody (Life Technologies), during 18–22 h at 4°C. After incubation, magnetic beads were washed four times with binding buffer and once with PBS. Beads containing exosomes were fixed in 2% glutaraldehyde and 1% tannic acid in 0.1M cacodylate buffer (pH 7.4) for 1 h. After two washes with cacodylate buffer, magnetic beads were incubated overnight in cacodylate buffer with 2% glutaraldehyde and then postfixed in 1% osmium tetroxide solution containing 1.5% of potassium ferricyanide for 2 h, rinsed in Milli-Q water, and dehydrated in a graded ethanol series. The exosomes bound to the Thermanox coverslips or to the magnetic beads were embedded in EMBED 812 (EM Sciences). Thin sections were cut perpendicular to the coverslips with a diamond knife, mounted on copper grids, and stained for 10 min each in Reynolds's lead citrate [51] and 0.5% aqueous uranyl acetate, then examined with a JEOL 100cx transmission electron microscope (JEOL, Tokyo, Japan).

### Scanning electron microscopy

Exosomes were pelleted from 100 mL of culture supernatants from A3.01 Nef/GFP or GFP cells as described above and the pellet was suspended in 100 µL of PBS. Aliquots of 30 µL were placed on 13-mm round coverslips previously treated with Biobond and allowed to adhere for 40 minutes. Exosomes were rinsed in PBS, fixed in 2% glutaraldehyde (EM Sciences) in PBS for 2 hours at room temperature and postfixed in 1% osmium tetroxide (EM Sciences) for 2 hours. Subsequently, the exosomes were rinsed in Milli-Q Water and incubated with a saturated solution of thiocarbohydrazide (EM Sciences) followed by 1% osmium tetroxide. This step was performed twice. The exosomes were dehydrated in a graded ethanol series and critically point-dried with liquid CO_2_ in a Bal-Tec-CPD 030 critical-point dryer (BAL-TEC, Balzers, Liechtenstein). The coverslips were mounted on aluminium stubs with silver paint (EM Sciences), and coated with gold in a Bal-Tec SCD 050 Sputter Coater (BAL-TEC). Samples were examined with a JEOL JSM- 6610LV scanning electron microscope (JEOL, Tokyo, Japan).

### HIV-1 infectivity assay

Exosomes were pelleted from cell culture supernatants as described above, washed with PBS and the amount of exosome proteins was determined using Pierce BCA protein assay kit (Thermo Scientific, Rockford, IL). Under these conditions approximately 4 µg of exosome proteins were recovered from 100 mL of conditioned media (2×10^5^ cells/mL cultured for 72 h). The pellet containing exosomes (approximately 4 µg of exosome proteins) was suspended in 4 mL of RPMI 1640 media containing 10% of exosome-free FBS and used for HIV-1 infectivity assays. For the assays, a 400 µL aliquot of media containing approximately 400 ng, 80 ng, 48 ng or 16 ng of exosome proteins was incubated for 1 h with 100 µL of conditioned media containing HIV-1 luciferase reporter particles (equivalent to ∼8 ng of p24). Thus the final concentrations of exosome proteins on infection assays were: 800 ng/mL, 160 ng/mL, 96 ng/mL or 32 ng/mL, as indicated in the figures. Viruses incubated with 400 µL of exosome-depleted culture media were used as control. After incubation these virus suspensions were added to 8×10^4^ MT4 cells and incubated for 4 h with agitation every 30 min. The cells were then washed, suspended in 500 µL of exosome-depleted RPMI 1640 media containing 10% FBS and cultivated for 48 h. After this period, the cells were washed with PBS, lysed and subjected to luciferase activity measurement using the Luciferase Assay System (Promega Corporation, Madison, WI) according to the manufacturer's protocol. The luciferase activity was measured using a Glomax^20/20^ Luminometer (Turner BioSystems, Sunnyvale, CA).

### Data analysis

All statistical analyses were performed using GraphPad Prism 5 software. Significance was evaluated by two-tailed paired Student's *t* test and P values are represented as: *, P<0.05; **, P<0.005, and ***, P<0.0005; NS, not significant.

## Results

### Characterization of CD4 and MHC-I downregulation in A3.01 T cells expressing Nef

**Figure 1:**
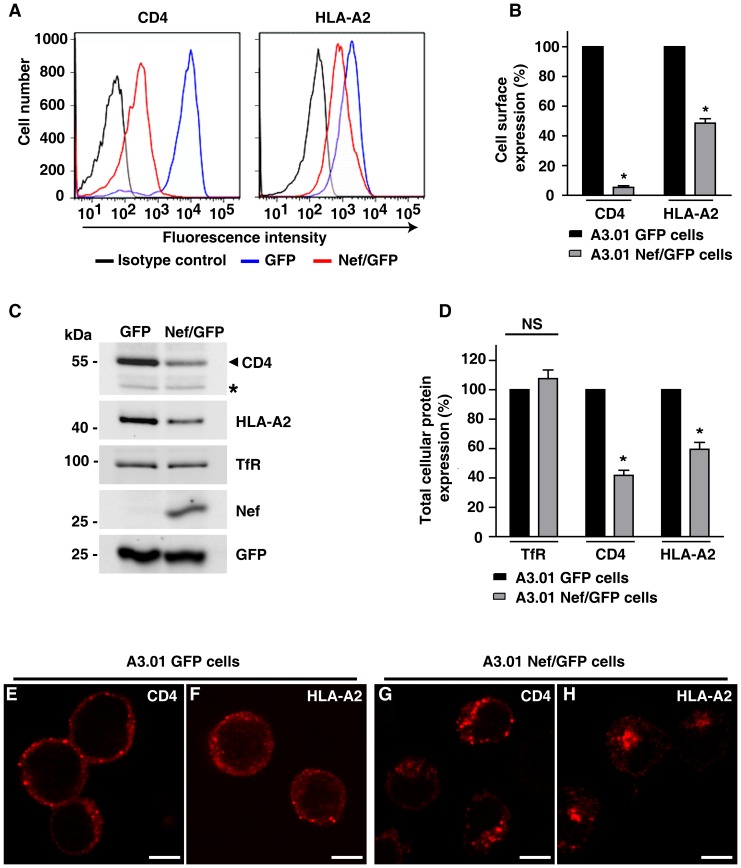
Characterization of T cells expressing GFP/Nef. CD4^+^ A3.01 T cells were transduced with retrovirus for expression of Nef and GFP (Nef/GFP) or GFP alone. In both cases, cells expressing GFP were selected with a cell sorter and cultured. (A) A3.01 Nef/GFP (red line) or GFP (blue line) cells were analyzed for cell surface expression of CD4 and HLA-A2 by FACS, as described in Materials and Methods. As negative controls, A3.01 Nef/GFP cells were labeled with the appropriated isotype control antibody (black line). (B) Bar graphs represent surface levels of CD4 or HLA-A2 (median values from FACS histogram plots) in Nef/GFP cells relative to levels in control GFP cells (set to 100%). Bars represent the means ± standard deviations of 3 independent experiments. Significance calculated by Student's t-test were as follows: *P<0.05. (C) Total cell extracts of Nef/GFP or GFP cells were analyzed by SDS-PAGE (10% gel) and western blot with the indicated antibodies. The CD4 antibody detects a nonspecific band (asterisk) that serves as an internal loading control. (D) The signal intensity of CD4, HLA-A2 and transferin receptor (TfR) for Nef/GFP and GFP cells was determined by densitometry using Image J software and were used to calculate the relative amount of these proteins in either Nef/GFP or GFP cells (set to 100%). Bars represent the means ± S.D. of normalized data from four independent experiments. The P-values calculated by Student's t-test using the raw data from gel densitometry were as follows: * P<0.05; NS, not significant. (E to H) A3.01 cells expressing GFP (E and F) or Nef and GFP (G and H) were adhered to coverslips coated with Biobond, fixed, permeabilized and stained with mouse monoclonal antibody to CD4 (E and G) or with rabbit polyclonal antibody to HLA-A2 (F and H) followed by Alexa-594-conjugated donkey anti-mouse IgG or Alexa-594-conjugated donkey anti-rabbit IgG (both shown in red channel). Cells were imaged by confocal laser scanning microscopy. Bars, 5 µm.

Downregulation of CD4 and MHC-I by Nef involves their redistribution to multivesicular bodies (MVBs). Considering that exosomes can derive from a subset of MVBs, we asked if Nef modifies the content of exosomes secreted by T cells and whether the secretory route contributes to CD4 and/or MHC-I downregulation. To test this, we transduced the A3.01 CD4^+^ T cells with bicistronic IRES-based retroviral vectors to express either Nef and GFP (A3.01 Nef/GFP) or GFP alone (A3.01 GFP), and GFP positive cells were separated by cell sorting. As expected, cell surface expression of CD4 and HLA-A2, a human MHC-I allotype sensitive to Nef, was reduced as a result of Nef expression. Quantitative analyses by flow cytometry showed that the cell surface levels of CD4 and HLA-A2 in A3.01 Nef/GFP cells were reduced by 94.6% (±2.06%) and 51.4% (±5.2%), respectively, compared to control A3.01 GFP cells (Fig. 1A and B). Analysis of total cell extracts by Western blot revealed that total levels of CD4 and HLA-A2 were also reduced in A3.01 Nef/GFP cells when compared to control cells expressing GFP alone (Fig. 1C and D).

To confirm delivery of CD4 and HLA-A2 to endosomes induced by Nef, the subcellular distribution of these proteins in GFP and Nef/GFP expressing cells was analyzed by confocal microscopy. In control A3.01 GFP cells, both CD4 and HLA-A2 are detected mainly at the cell periphery (Fig. 1E and F). In contrast, A3.01 Nef/GFP cells showed a weak CD4 and HLA-A2 labeling at the cell periphery that was accompanied by an increased localization of these proteins in intracellular structures (Fig. 1G and H). In Nef/GFP cells, both CD4 and HLA-A2 are partially localized to vesicles containing the ESCRT-0 subunit HRS (see Fig. S1A–C and D–F) and lysobisphosphatidic acid – LBPA (Fig. S1G–I and J–L), an endosome specific phospholipid that is abundant in intraluminal membranes of MVBs [52]–[54]. Thus in the presence of Nef, CD4 and HLA-A2 are redistributed from the plasma membrane to endosomal compartments containing HRS and/or LBPA (Fig. S1), which are both involved in MVB biogenesis [55]–[57]. Together, these results confirmed that the Nef protein expressed in A3.01 Nef/GFP cells is functional and that these cells constitute tools to study the effects of Nef in T cell-derived exosomes.

### Extracellular vesicles released from A3.01 T cells expressing Nef/GFP or GFP are similar in average size and morphology

**Figure 2:**
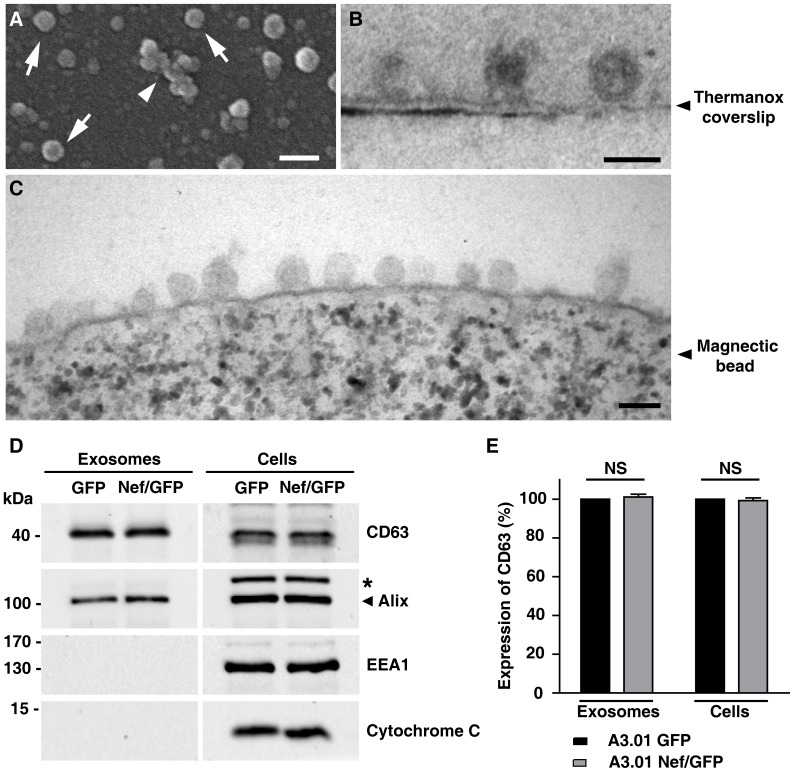
Characterization of exosomes released from A3.01 T cells expressing GFP or Nef/GFP. (A) SEM analysis of exosomes released from A3.01 T GFP cells. Exosomes are seen isolated as vesicles of ∼100 nm (arrows), or associated as larger amorphous structures (arrow head). Bar, 0.2 µm (B) TEM analysis of A3.01 GFP T cells exosomes showing elliptical structures enclosed by a lipid-bilayer. Exosomes were isolated, placed in Biobond coated Thermanox coverlips, fixed and analyzed by TEM as described in the material and methods section. Bar, 0.1 µm. (C) Exosomes isolated by ultracentrifugation were captured by magnetic beads coated with anti-CD63 antibody, fixed and analyzed by TEM as described in the material and methods. Bar, 0.1 µm (D) Exosomes released from A3.01 T cells expressing GFP or Nef/GFP were isolated by ultracentrifugation from equal amounts of culture media conditioned by equivalent number of cells. Exosome and total cell lysates were subjected to SDS-PAGE and western blot with antibodies to CD63 and Alix (exosomal markers), EEA1 and cytochrome C. The antibody to Alix detects a nonspecific band (asterisk) that served as an internal loading control. (E) The CD63 signal for each condition shown in panel C was determined and used to calculate the relative amount of CD63 in either exosomes or cell lysates from Nef/GFP cells relative to GFP cells (100%). Bars represent the means ± standard deviations of normalized data from 3 independent experiments and P-values were calculated using raw data from densitometry using Student's t-test; NS, not significant.

To investigate the effects of Nef on exosomes, we isolated these extracellular vesicles released from A3.01 Nef/GFP and A3.01 GFP lymphocytes. Cells were cultured for 72 h in exosome-free culture media, and the exosomes released were harvested from the conditioned media by ultracentrifugation. Scanning electron microscopy (SEM) analyses of the pelleted material from A3.01 GFP cells revealed vesicular structures (Fig. 2A, arrows) that ranged from 40 nm to 114 nm in diameter (see Fig. S2) and were often associated to each other as larger amorphous structures (Fig. 2A, arrowhead). Similar results were observed for exosomes isolated from A3.01 Nef/GFP cells (Fig. S2). Transmission electron microscopy (TEM) analyses of exosomal fractions revealed elliptical structures enclosed by a lipid-bilayer with approximate diameters of 100 nm (Fig. 2B). Similar results were obtained by TEM analyses of exosomes captured from exosomal fractions by magnetic beads coated with anti-CD63 antibody (Fig. 2C). Together, the electron microscopy analyses indicated that Nef expression does not modify the morphology or average size of the exosomes.

To verify the composition of the exosomes and rule out the presence of non-exosomal membranes, the pellets from ultracentrifugation were analyzed by Western blot. Both CD63 and Alix, which are well-established markers for exosomes [41], [58], were enriched in our preparations (Fig. 2D). In addition, the purity of these preparations was confirmed by the absence of EEA1, a peripheral early endosomal protein, and cytochrome C, a mitochondrial protein (Fig. 2D). From these data, we concluded that vesicles released from our GFP and Nef/GFP lymphocytes are typical exosomes, free from cellular contaminants. We did not detect significant differences in the total amount of exosomal proteins released by the same number of A3.01 GFP and Nef/GFP lymphocytes (Fig. 2E), suggesting that Nef may not interfere with the amount of exosomes released by those cells.

### Nef decreases the expression of CD4 and HLA-A2 in exosomes

**Figure 3:**
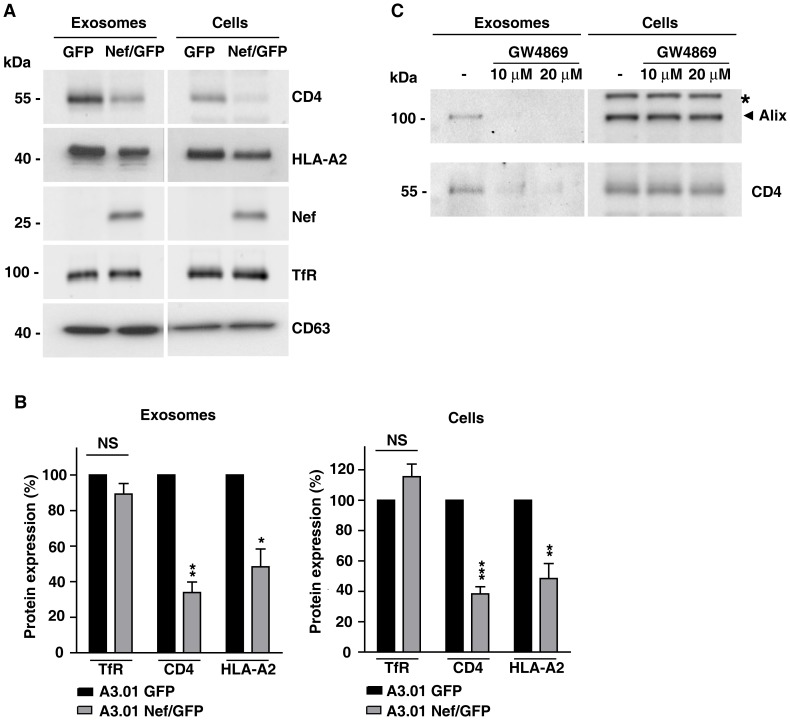
Nef reduces expression of CD4 and HLA-A2 in exosomes from CD4^+^ T cells. (A) Exosomes released from equal numbers of A3.01 T cells expressing GFP or Nef/GFP were isolated from culture supernatant after 72 h of culture and equivalent amounts of exosome lysates or cell lysates were western blotted with antibodies to CD4, HLA-A2, Nef, TfR and CD63. (B) The TfR, CD4 and HLA-A2 signals for each condition shown in panel A was determined by densitometry and used to calculate the relative amount of these proteins in either exosomes (left panel) or total cell (right panel) lysates from Nef/GFP relative to GFP cells (100%). Bars represent the means ± standard deviations (n = 3) of normalized data. P-values calculated by Student's t-test using the raw data from densitometry analysis were as follows: * P<0.05; **, P<0.005, and ***, P<0.0005; NS, not significant. (C) Inhibition of CD4 secretion in exosomes by GW4869. A3.01 GFP cells were cultivated in absence or presence of 10 µM or 20 µM of GW4869 for 24 h. After treatment, conditioned medium were collected and exosomes were purified as described in material and methods. Exosomal and cellular proteins were analyzed by SDS-PAGE (6% gel) and western blot with antibodies to Alix or CD4. A nonspecific band detected with anti-Alix antibody is indicated with an asterisk. Note that treatment with GW4869 reduced the amount of both Alix and CD4 recovered in the exosome fractions. The results shown are representative of three independent experiments.

Since Nef promotes its own secretion via exosomes and depletes intracellular levels of various transmembrane proteins, the ability of Nef to alter the protein composition of exosomes was examined. Western blot analysis of exosomes released from A3.01 GFP lymphocytes revealed the presence of CD4 (Fig. 3A), indicating that this co-receptor is a component of exosomes derived from CD4^+^ T lymphocytes. In addition, Nef was detected in exosomes derived from A3.01 Nef/GFP cells (Fig. 3A). Interestingly, we observed a decrease of approximately 66% in the amount of CD4 present in exosomes from A3.01 Nef/GFP cells compared with exosomes from A3.01 GFP cells (Fig. 3A and B). Nef also reduced the amount of HLA-A2 in exosomes, by approximately 50% (Fig. 3A and B). Noticeably, the amount of transferrin receptor (TfR), a distinct cell surface receptor, did not change in response to Nef expression (Fig. 3A and B). It was recently reported that surface TfR levels are unaffected by HIV-1 Nef and elevated by SIV Nef (Koppensteiner, et al., 2014). Also, there were no significant differences in the levels of the exosomal marker CD63, as a consequence of Nef expression.

To further confirm that the CD4 detected in our experiments is a component of exosomes secreted from CD4^+^ T cells, and not simply derived from shedding of plasma membrane fragments, we treated cells with the neutral sphingomyelinase (nSMase) inhibitor GW4869 that is a well-known inhibitor of exosome release [59]–[63]. As expected, treatment with GW4869, led to a marked reduction in total exosome release, as indicated by the decrease of Alix recovered in the exosome fractions (Fig. 3C). After GW4869 treatment the levels of CD4 in the exosome fractions were also greatly reduced, without a significant change in the amount of CD4 expressed in cells (Fig. 3C). Taken together, these results demonstrate that Nef has the ability to reduce the amounts of CD4 and MHC-I secreted in exosomes, and that depletion of cell surface and total cell levels of CD4 and MHC-I by Nef is not due to increased secretion of these transmembrane proteins via exosomes. Treatment of A3.01 Nef/GFP cells with bafilomycin A1, an inhibitor of acidification and protein degradation in lysosomes, led to the recovery of intracellular levels of CD4 and HLA-A2, without increasing the levels of Nef (see Fig. S3). This indicates that, differently from CD4 and HLA-A2, Nef itself is not targeted to ILVs of MVBs destined for lysosomes.

### Exosomes containing CD4 inhibit HIV-1 infection

**Figure 4:**
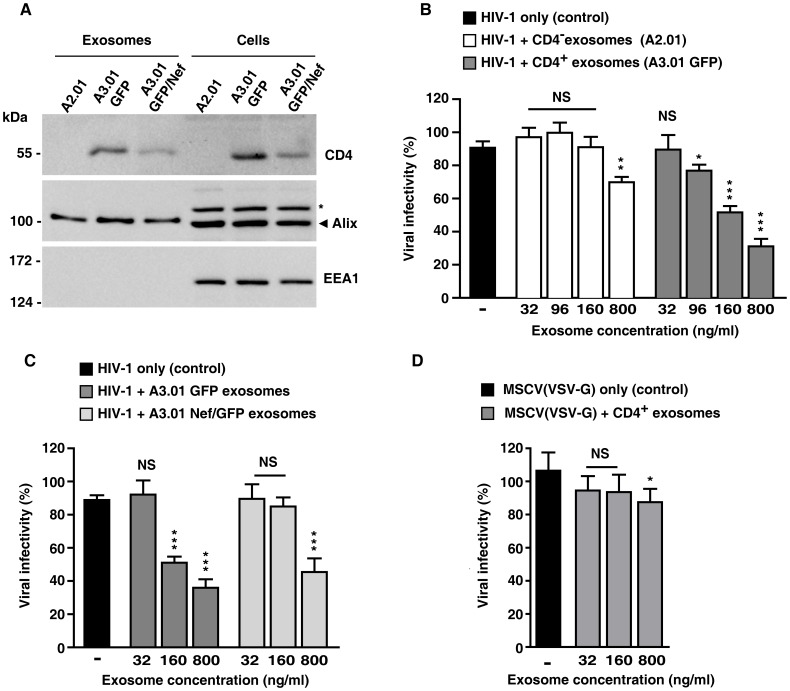
Exosomes containing CD4 reduce HIV-1 infection. (A) Exosomes derived from A2.01, A3.01 GFP and A3.01 GFP/Nef cells were purified from 100 mL cell culture supernatants by ultracentrifugation. Proteins in exosome and cell lysates were analyzed by Western blot with antibodies to CD4, Alix and EEA1 (an early endosome protein absent in exosomes). An asterisk indicates a nonspecific band recognized by the anti-Alix antibody. (B) Aliquots of HIV-1 Luc^+^ particles (equivalent to 8 ng of p24) were incubated for 1 h with exosomes isolated from A2.01 or A3.01 GFP cell supernatants. The final concentrations of exosomes protein used in the assays are indicated. These HIV-1-exosome preparations were then used to infect MT4 cells. Cells infected with reporter viruses pre-incubated with exosome-free cell culture media were used as positive control of infection. The infectivity levels were evaluated by luciferase activity in MT4 cell lysates, and expressed as percentage of the levels in cells infected with HIV-1 Luc^+^ in the absence of exosomes (control), with the highest level of luciferase activity in control conditions set to 100%. The data represent the means ± standard deviations from three independent experiments. (C) Infection assays were repeated in MT4 cells using HIV-1 Luc^+^ particles pre-incubated with exosomes from A3.01 GFP and A3.01 Nef/GFP cells, as described above. Bar graph represents luciferase activity in MT4 cells infected with HIV-1 Luc^+^ in the presence of A3.01 GFP and A3.01 Nef/GFP exosomes, relative to levels in MT4 cells infected with HIV-1 Luc^+^ in the absence of exosomes (100%). The data represent the means ± standard deviations from three independent experiments. (D) Infection assays were performed in MT4 cells using a VSV-G pseudotyped MSCV that encodes GFP. Aliquots of VSV-G-MSCV(GFP) particles were incubated for 1 h with exosomes isolated from A3.01 GFP cell supernatants. The final concentrations of total exosome proteins used in the assays are indicated. The MSCV-exosome preparations were used to infect MT4 cells and cells infected with reporter viruses pre-incubated with exosome-free cell culture media were used as positive control of infection. The infectivity levels were evaluated by intensity of GFP fluorescence in cells by FACS analysis. Bar graph represent the levels of GFP fluorescence (median values from FACS histogram plots) from cells transduced with VSV-G-MSCV(GFP) pre-incubated with CD4^+^ exosomes, relative to cells transduced with VSV-G-MSCV(GFP) in the absence of exosomes (100%). P-values were calculated using the Student's t-test: * P<0.05; **, P<0.005, and ***, p<0.0005; NS, not significant.

Since we observed that exosomes released from T cells contain large amounts of CD4, we hypothesized that these CD4^+^ exosomes could compete with host cell surface CD4 for gp120 interaction and hinder HIV infection. To test this, HIV-1 infection assays were performed in the presence of purified exosomes. Thus, NL4.3 luciferase reporter viruses (HIV-1 Luc^+^) were pre-incubated with increasing concentrations of exosomes obtained from CD4^+^ A3.01 lymphocytes or from a variant cell line (A2.01) lacking CD4 (Fig. 4A), and these viruses were then used to infect lymphocytic CD4^+^ T cells (MT4). As a positive infection control, MT4 cells were infected with HIV-1 Luc^+^ in absence of exosomes. As expected infection of MT4 cells with HIV-1 Luc^+^ resulted in detectable luciferase activity (Figure 4B). Remarkably, a dose-dependent decrease in luciferase activity was seen in MT4 cells that were infected HIV-1 Luc^+^ particles that were pre-incubated with increasing concentrations of CD4^+^ exosomes (Figure 4B). Notably, the capacity of HIV-1 Luc^+^ to infect MT4 cells was largely unaffected when virus particles were pre-incubated with exosomes that do not contain CD4 (Fig. 4B). A reduction in infection by exosomos without CD4 was only detectable when HIV-1 Luc^+^ particles were pre-incubated with a high concentration of these exosomes, a concentration 20-fold higher than that found in conditioned culture media.

Since expression of CD4 was reduced in exosomes from Nef-expressing cells, similar experiments were performed to test the capacity of HIV-1 Luc^+^ to infect MT4 cells in presence of exosomes from A3.01 Nef/GFP lymphocytes. Exosomes purified from cells expressing Nef presented a reduced capacity to interfere with HIV-1 infection, as higher concentrations of these exosomes were necessary to inhibit infection compared to exosomes from GFP cells (Fig. 4C). We conclude that exosomes released by CD4^+^ T cells inhibit HIV-1 infection and that Nef hinders this inhibitory effect by reducing the amount of CD4 on the surface of exosomes, thus promoting viral spreading. Infection of MT-4 cells by VSV-G pseudotyped MSCV encoding GFP, was not affected by pre-incubation with CD4^+^ exossomes at concentrations that were inhibitory for Env-dependent HIV-1 infection (Fig. 4D). At the highest concentration of CD4+ exosomes (∼800 ng/ml) a small (∼10%) inhibition of VSVG-MSCV infection was detected. However, at this concentration of exosomes HIV-1 infection was inhibited by ∼75% suggesting that these exosomes have the specific ability to block Env-dependent viruses. Together, the results indicate that CD4^+^ exosomes are detrimental to HIV-1 infection because of unproductive Env-mediated interaction with CD4 molecules exposed on the surface of exosomes.

## Discussion

Recently, several studies have aimed to elucidate the role of exosomes in intercellular communication, where they may act as long-range messenger particles. The results of the present study reveal that Nef modifies the composition of exosomes released by T cells, reducing the levels of both CD4 and MHC-I molecules in these extracellular vesicles. It has been previously shown that HIV-1 Nef induces the release of exosomes and is itself secreted via these vesicles [39], [40]. Since Nef targets CD4 and MHC-I to ILVs of MVBs [21], [29], [30] and Nef is released in exosomes, which may originate in these ILVs, we tested whether this secretory route could contribute to deplete cellular levels of CD4 and/or MHC-I as previously postulated [64]. Interestingly, the results shown here indicate the opposite, the amount of CD4 and MHC-I released in exosomes is actually reduced as a consequence of Nef expression. Therefore this route is unlikely to contribute to their downregulation.

We showed that inhibition of lysosomal activity in A3.01 Nef/GFP cells leads to recovery of intracellular levels of CD4 and HLA-A2, without increasing the levels of Nef, as was previously observed for HeLa cells [30]. We conclude that in contrast to CD4 and MHC-I, Nef is not delivered to lysosomes. Together, these data suggest that Nef escapes the MVB pathway leading to lysosomes that is taken by CD4 and MHC-I molecules. Instead, Nef may be recruited to a subset of MVBs that fuse with the plasma membrane or alternatively to domains at the plasma membrane enriched in exosomal markers shown to exist in certain T cell lines, which are competent for outward vesicle budding [65].

In addition to being secreted in exosomes, HIV-1 Nef was previously shown to induce the release of these extracellular vesicles [39], [40]. Here we confirm that Nef is secreted in exosomes, however we did not observe significant changes in the amount of exosomes released by A3.01 lymphocytes as a result of Nef expression. The reasons for this discrepancy are not apparent, but they may be related to differences in the cell lines used or the levels of Nef expression resulting from different expression systems. In these previous studies, Nef expression was achieved by plasmid electroporation [39] or by rapid pharmacological activation of a Nef-fusion protein originally expressed in an inactive form [40], whereas here retroviral transduction was used.

CD4 is a receptor molecule required for HIV-1 infection and its removal from the surface of infected cells by Nef is thought to prevent multiple rounds of re-infection [3]. Furthermore, the presence of CD4 in the secretory pathway and on the surface of infected cells leads to the formation of CD4-Env complexes that compromise trafficking of viral envelope proteins and thus reduce viral infectivity [66]. Our results showed that the presence of CD4^+^ exosomes also decreases the efficiency of HIV-1 infection *in vitro*.

This inhibition is likely due to the presence of CD4 molecules in these extracellular vesicles, since equivalent amounts of exosomes prepared from a T cell line that does not express CD4 were not able to inhibit HIV-1 infection. We postulate that inhibition is caused by interaction between Env proteins on the virus envelope and the CD4 ectodomain displayed by these exosomes. In fact, we show that infection by an Env-independent virus is not inhibited by CD4^+^ exosomes. The interaction of HIV-1 with CD4^+^ exosomes would result in a reduction of unbound Env proteins available to interact with CD4 molecules on the surface of host cells, thus hindering infection. Previous studies have shown that soluble forms of the CD4 receptor (sCD4) can inhibit virus infection and competition between sCD4 and cellular CD4 was proposed as the major mechanism of HIV-1 inhibition by sCD4 [67], [68].

Most importantly, our results showed that exosomes released by CD4^+^ T cells expressing Nef were not able to efficiently reduce HIV-1 infection, probably due to the decreased expression of CD4 in these exosomes. These data reveal a potentially novel role for Nef in facilitating viral spread by reducing expression of CD4 in exosomes and thus promoting HIV-1 infection. Exosomes are thought to be present in various bodily fluids, including blood plasma, in physiological conditions [69], [70]. Thus we propose that the presence of CD4^+^ exosomes at the sites of infection could be detrimental to efficient viral spreading by reducing the population of disengaged/free viral particles. Interestingly, it has recently been shown that extracellular vesicles are released by T cells in a polarized manner and are trapped and accumulate in the immune synaptic cleft. Furthermore, HIV-1-Gag containing viral like particles (VLPs) are also released into these sites of cell-cell contact [71], [72]. Accordingly, preventing release of CD4+ exosomes at the site of the HIV-1 virological synapse could facilitate infection of the target cell.

The present study also revealed that Nef reduces the amount of MHC-I molecules in exosomes released by T cells; however, the biological role of MHC-I exosomal depletion by Nef was not assessed here. Since peptide-MHC-I complexes displayed on exosomes can be recognized by immune receptors and trigger immune responses ([42] and references therein), it is tempting to speculate that, by removing of MHC-I molecules from exosomes released from infected cells, Nef may compromise long-range activation of the immune system. In fact, there is strong evidence that the ability of Nef to compromise antigen presentation by MHC-I allows HIV-infected cells to escape recognition by cytotoxic T lymphocytes [73] and that this activity may be important for pathogenesis of HIV-1 infection *in vivo* [15]. Interestingly, a recent study by Näslund et al., [74] showed that pre-exposure of dendritic cells to exosomes derived from human breast milk of healthy donors, reduces infection by HIV-1. Since this protective capacity varied according to the source of exosomes [74], exosomal composition is likely to play an essential role in this process. Further studies are necessary to fully access the implications of the changes in exosomal composition promoted by Nef in HIV-1 infection.

## Supporting Information

Figure S1 **Nef redistributes CD4 and HLA-A2 to endosomes containing HRS and/or LBPA.** (A to D) A3.01 Nef/GFP T cells were attached to coverslips coated with Biobond, fixed, permeabilized and double stained with: (A) mouse monoclonal antibody against CD4 and rabbit polyclonal antibody to HRS, followed by donkey anti mouse IgG conjugated to Alexa-594 (red channel) and donkey anti rabbit IgG conjugated to Alexa-647 (green channel); (B) rabbit polyclonal antibody to HLA-A2 and mouse monoclonal antibody to HRS, followed by donkey anti rabbit IgG conjugated to Alexa-594 (red channel) and donkey anti mouse IgG conjugated to Alexa-647 (green channel); (C) mouse IgG2a monoclonal antibody to CD4 and mouse IgG1 monoclonal antibody to LBPA, followed by Alexa-594-conjugated goat polyclonal antibody specific to mouse IgG2a isotype (red channel) and Alexa 647-conjugated goat polyclonal antibody specific to mouse IgG1 isotype (green channel); (D) rabbit polyclonal antibody to HLA-A2 and mouse monoclonal antibody to LBPA, followed by donkey anti rabbit IgG conjugated to Alexa-594 (red channel) and donkey anti mouse IgG conjugated to Alexa-647 (green channel). Cells were imaged by confocal laser scanning microscopy. Yellow in the merged images indicates colocalization. Bar, 5 µm. The insets represent the boxed areas at a magnification of ×2.5.
(TIF)

Figure S2 **The Nef expression does not modify the average size of exosomes released by A3.01 T cells.** Exosomes from A3.01 GFP and A3.01 Nef/GFP cells were isolated and prepared for SEM analyses as described in material and methods. The diameter of 100 isolated exosomes from GFP and Nef/GFP cells was determined from SEM images (as shown in Fig. 2) using ImageJ software. The graph shows the percentage of exosomes with diameters corresponding to: 30–50 nm, 51–100 nm or larger than 100 nm for either GFP or Nef/GFP cells. The data represent the means ± standard deviations from three independent experiments. P-values were calculated using the Student's t-test. NS, not significant.
(TIF)

Figure S3 **Nef targets CD4 and HLA-A2 to lysosomes but escapes from this degradative pathway.** Nef/GFP A3.01 cells were incubated in the absence (−) or presence of 1 µM bafilomycin A1 for the different periods indicated in the figure. Total cell extracts were analyzed by SDS-PAGE and western blot with the indicated antibodies. The CD4 and Alix antibodies detect a nonspecific band (asterisk) that serves as an internal loading control. Molecular mass (in kDa) markers are indicated on the left. The results shown are representative of three independent experiments. Notice that incubation with bafilomycin A1 leads to a time-dependent increase in the levels CD4 and HLA-A2 in A3.01 Nef cells, whereas the levels of Nef do not increase.
(TIF)
