# Supplementary figures and images for: Nef Neutralizes the Ability of Exosomes from CD4+ T Cells to Act as Decoys during HIV-1 Infection
Source: PLoS One. 2014 Nov 25;9(11):e113691. doi: 10.1371/journal.pone.0113691 (PMC4244142; doi:10.1371/journal.pone.0113691)

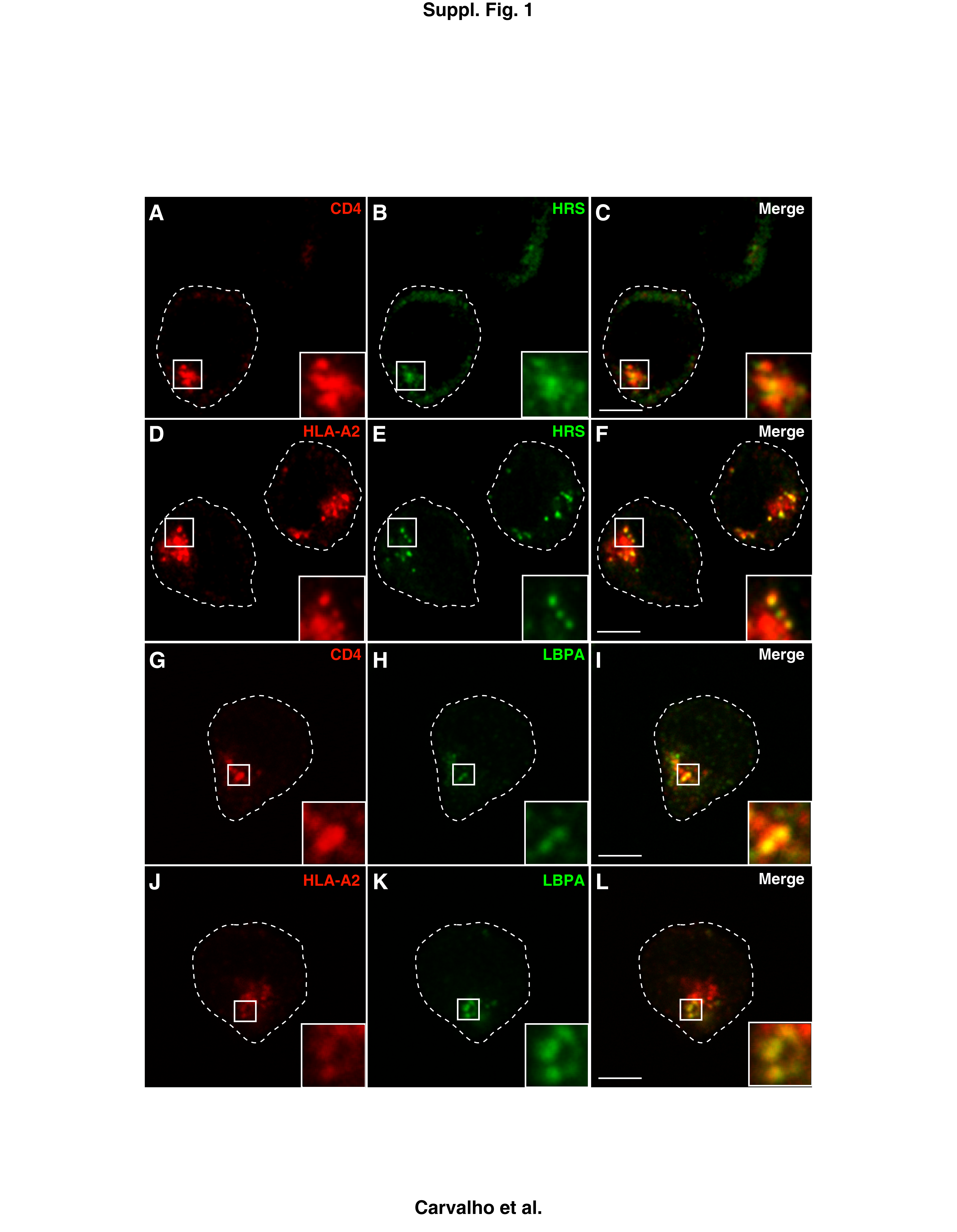

Supplement: Figure S1 — Nef redistributes CD4 and HLA-A2 to endosomes containing HRS and/or LBPA. (A to D) A3.01 Nef/GFP T cells were attached to coverslips coated with Biobond, fixed, permeabilized and double stained with: (A) mouse monoclonal antibody against CD4 and rabbit polyclonal antibody to HRS, followed by donkey anti mouse IgG conjugated to Alexa-594 (red channel) and donkey anti rabbit IgG conjugated to Alexa-647 (green channel); (B) rabbit polyclonal antibody to HLA-A2 and mouse monoclonal antibody to HRS, followed by donkey anti rabbit IgG conjugated to Alexa-594 (red channel) and donkey anti mouse IgG conjugated to Alexa-647 (green channel); (C) mouse IgG2a monoclonal antibody to CD4 and mouse IgG1 monoclonal antibody to LBPA, followed by Alexa-594-conjugated goat polyclonal antibody specific to mouse IgG2a isotype (red channel) and Alexa 647-conjugated goat polyclonal antibody specific to mouse IgG1 isotype (green channel); (D) rabbit polyclonal antibody to HLA-A2 and mouse monoclonal antibody to LBPA, followed by donkey anti rabbit IgG conjugated to Alexa-594 (red channel) and donkey anti mouse IgG conjugated to Alexa-647 (green channel). Cells were imaged by confocal laser scanning microscopy. Yellow in the merged images indicates colocalization. Bar, 5 µm. The insets represent the boxed areas at a magnification of ×2.5. (TIF) [file pone.0113691.s001.tif]

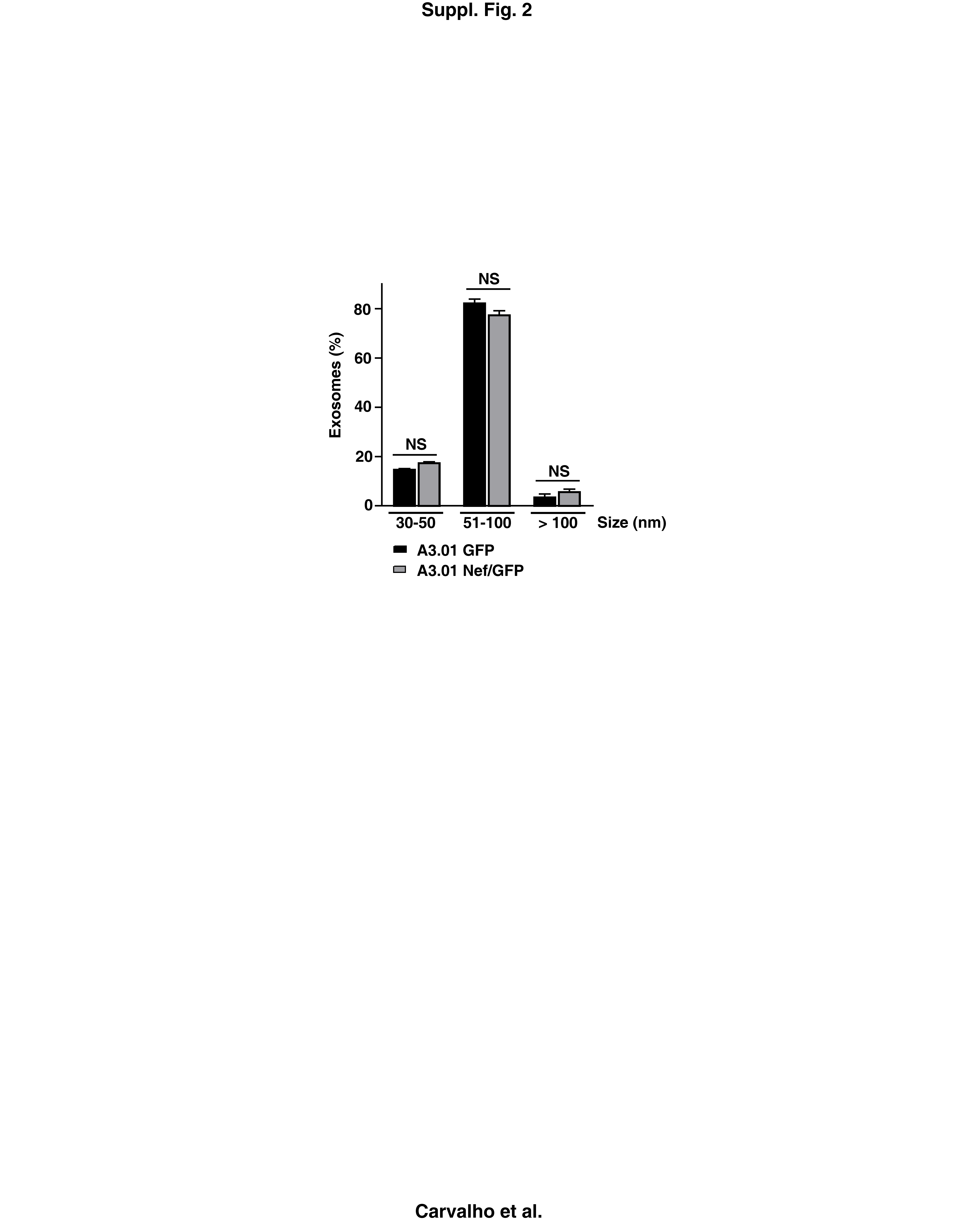

Supplement: Figure S2 — The Nef expression does not modify the average size of exosomes released by A3.01 T cells. Exosomes from A3.01 GFP and A3.01 Nef/GFP cells were isolated and prepared for SEM analyses as described in material and methods. The diameter of 100 isolated exosomes from GFP and Nef/GFP cells was determined from SEM images (as shown in Fig. 2) using ImageJ software. The graph shows the percentage of exosomes with diameters corresponding to: 30–50 nm, 51–100 nm or larger than 100 nm for either GFP or Nef/GFP cells. The data represent the means ± standard deviations from three independent experiments. P-values were calculated using the Student's t-test. NS, not significant. (TIF) [file pone.0113691.s002.tif]

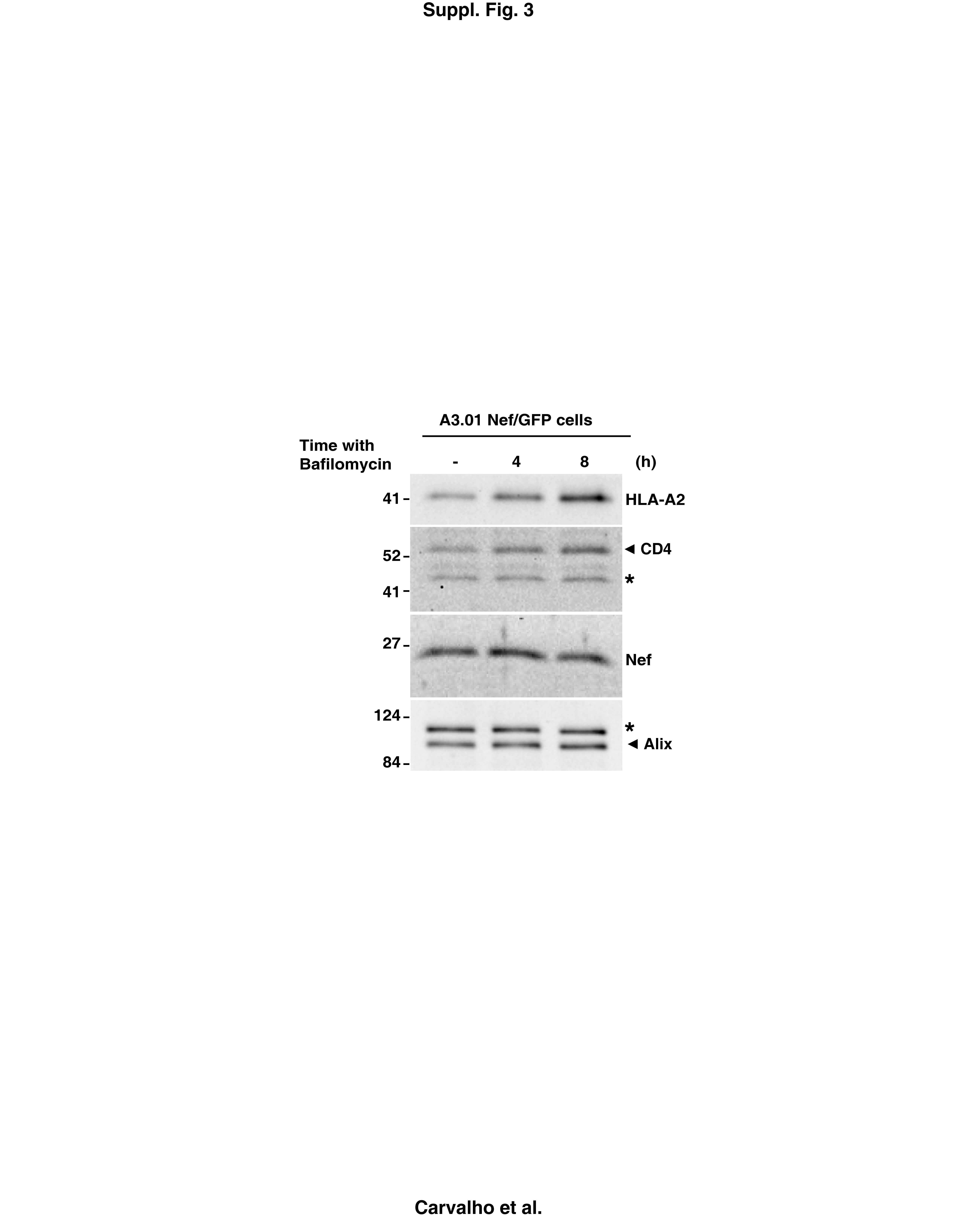

Supplement: Figure S3 — Nef targets CD4 and HLA-A2 to lysosomes but escapes from this degradative pathway. Nef/GFP A3.01 cells were incubated in the absence (−) or presence of 1 µM bafilomycin A1 for the different periods indicated in the figure. Total cell extracts were analyzed by SDS-PAGE and western blot with the indicated antibodies. The CD4 and Alix antibodies detect a nonspecific band (asterisk) that serves as an internal loading control. Molecular mass (in kDa) markers are indicated on the left. The results shown are representative of three independent experiments. Notice that incubation with bafilomycin A1 leads to a time-dependent increase in the levels CD4 and HLA-A2 in A3.01 Nef cells, whereas the levels of Nef do not increase. (TIF) [file pone.0113691.s003.tif]
